# Low Serum Concentration of Obestatin as a Predictor of Mortality in Maintenance Hemodialysis Patients

**DOI:** 10.1155/2013/796586

**Published:** 2013-09-12

**Authors:** Ilia Beberashvili, Inna Sinuani, Ada Azar, Hadas Kadoshi, Gregory Shapiro, Leonid Feldman, Judith Sandbank, Zhan Averbukh

**Affiliations:** ^1^Nephrology Division, Assaf Harofeh Medical Center, Affiliated to Sackler Faculty of Medicine, Tel Aviv University, 70300 Zerifin, Israel; ^2^Pathology Department, Assaf Harofeh Medical Center, Affiliated to Sackler Faculty of Medicine, Tel Aviv University, 70300 Zerifin, Israel; ^3^Nutrition Department, Assaf Harofeh Medical Center, Affiliated to Sackler Faculty of Medicine, Tel Aviv University, 70300 Zerifin, Israel

## Abstract

Obestatin, a proposed anorexigenic gut hormone, has been shown to have a number of beneficial cardiotropic effects in experimental studies. We hypothesized that obestatin alteration in hemodialysis patients may link to clinical outcomes. This cross-sectional study with prospective followup for almost 4 years was performed on 94 prevalent hemodialysis patients. Obestatin, leptin, proinflammatory cytokines (tumor necrosis factor-**α** [TNF-**α**], interleukin-6, and various nutritional markers were measured. Patients with low obestatin levels, defined as a level less than median, had a worse all-cause mortality and cardiovascular mortality. The crude all-cause (HR 2.23, 95% CI 1.17 to 4.24) and cardiovascular mortality hazard ratios (HR 4.03, 95% CI 1.27 to 12.76) in these patients continued to be significant after adjustment for various confounders for all-cause mortality. Across the four obestatin-TNF-**α** categories, the group with low obestatin and high TNF-**α** (above median level) exhibited a worse outcome in both all-cause mortality and cardiovascular mortality. Clinical characteristics of patients in low obestatin high TNF-**α** group did not differ from other obestatin-TNF-**α** categorized groups. In summary, low serum obestatin concentration is an independent predictor of mortality in prevalent hemodialysis patients. Novel interactions were observed between obestatin and TNF-**α**, which were associated with mortality risk, especially those due to cardiovascular causes.

## 1. Introduction

Obestatin, an anorexigenic gut hormone, is a 23 amino acid peptide derived from the C terminal portion of the 117 amino-acid preproghrelin precursor. It was recently identified by Zhang et al. [1] using bioinformatic analyses of the preproghrelin genomic sequence in different species. Obestatin was proposed as the natural ligand of the G-protein-coupled receptor 39 (GPR39), a member of the G-protein-coupled receptor family which also includes the receptor for the hunger hormone, ghrelin. Obestatin was therefore claimed to be a physiological opponent of acylated ghrelin [2]. Treatment of rodents with obestatin suppresses food intake [3], inhibits jejunal contractions, and decreases body weight [1, 3]. However, soon after its discovery, obestatin became a very controversial peptide. Several following studies have questioned the inhibitory effects of obestatin on food intake and gastrointestinal motility [4] and its ability to represent the cognate ligand for the GPR39 receptor [5]. 

Recent accumulated experimental evidence suggests a protective cardiovascular effect of obestatin which may be comparable to that afforded by ghrelin [6]. The protective effects of obestatin on acute ischemia-reperfusion injury in isolated rat hearts and its antiapoptotic effects in cultured rat cardiomyocytes were recently demonstrated by Alloatti et al. [7]. Obestatin has been shown to prevent apoptosis in both rodent beta cells and human pancreatic islets by interacting with specific mechanisms proposed as integral components of the antiapoptotic cascades that are involved in myocardial protection [6]. Tumor necrosis factor-*α* stimulation together with obestatin treatment decreased vascular cell adhesion molecule-1 (VCAM-1) expression in endothelial cells cultures, suggesting that obestatin may modulate atherogenesis processes [8]. 

To date, little is known about obestatin levels and its behavior in maintenance hemodialysis (HD) patients. Serum obestatin levels in the end stage kidney disease patients were significantly higher compared to that of controls in one small cross-sectional study [9]. However, this finding was not confirmed by other studies with the same sample size and design [10, 11]. No direct relations between obestatin and the patients' food intake or appetite in a hemodialysis population have been found [10]. The association between obestatin and BMI in end stage kidney disease patients is also controversial [9–11]. Additionally, there has been no study focusing on the relationship between serum obestatin levels and the occurrence of clinically evident cardiovascular and all-cause mortality in HD patients. Studies on obestatin levels in patients with end stage kidney disease may be of interest because of its possible anorexigenic profile that can lead to protein-energy wasting, as well as the participation of obestatin in atherogenesis. Both pathways together or separately may predispose the patients to adverse cardiovascular outcomes and consequently to higher mortality rates. 

To assess the association of baseline obestatin with all-cause and cardiovascular mortality, we conducted a prospective cohort study in prevalent HD patients. In addition, we tested whether the associations of obestatin with other cytokines (tumor necrosis factor *α* (TNF-*α*), interleukin-6 (IL-6), leptin) play a role in clinical outcome in our cohort.

## 2. Materials and Methods

### 2.1. Study Population

This prospective observational study was approved by the Ethics Committee of Assaf Harofeh Medical Center (Zerifin, Israel). Informed consent was obtained before any trial-related activities. Patients were eligible for study entry if they had been on HD therapy for at least 3 months and were 18 years or older, with no clinically active cardiovascular or infectious diseases on entry. In total, 94 patients (58 men and 36 women) with a mean age of 64.8 ± 11.5 years were included in the study. All the patients received maintenance hemodialysis treatment at our outpatient HD clinic. The study population has been described in more detail in a recent publication [12]. From the 101 patients included in the previous cohort, the analysis presented here comprises the 94 patients for whom data on serum obestatin and TNF-*α* levels were available. In total, the study period extended for 45.8 ± 26.7 months (interquartile range 19.0–75.0 months). During this period, 39 patients (41.5%) died (the main causes of death were sepsis (16 of 39 patients; 41.0%) and cardiovascular diseases (15 of 39 patients; 38.5%])); 14 patients (20.6%) underwent kidney transplantation; 3 patients (4.4%) changed dialysis modality; and 10 patients (14.7%) transferred to other hemodialysis units. Thus, 27 patients were removed from the study from the time of their transplantation or from when they transferred to another hemodialysis unit. 

Information on the patient's past cardiovascular diseases (cerebral vascular, peripheral vascular, and heart disease) was obtained from a detailed medical history. Cardiovascular disease was defined as myocardial infarction (MI), requiring coronary artery procedures such as angioplasty or surgery, cerebral vascular accident (CVA), or peripheral vascular disease (PVD), requiring angioplasty, bypass, or amputation. Cardiovascular mortality was defined as death resulting from coronary heart disease, sudden death, stroke, or complicated peripheral vascular disease. 

### 2.2. Dietary Intake

A continuous 3-day dietary history (including a dialysis day, a weekend day, and a nondialysis day) was recorded in a self-completed food diary. The methods used for collecting the dietary recalls were the same as was those described by Bross et al. [13]. Dietary energy and protein intake were calculated and normalized for adjusted body weight. 

Dietary protein intake was also estimated by calculating normalized protein nitrogen appearance (nPNA) from the patient's urea generation rate by using urea kinetics modeling, the single-pool model [14]. 

### 2.3. Anthropometric Measurements

BMI, triceps skinfold thickness (TSF), midarm circumference (MAC), and calculated mid arm muscle circumference (MAMC) were measured for the anthropometric variables. The midweek, postdialysis weight was used for evaluation of BMI according to K/DOQI guideline recommendations. MAMC was estimated as follows:

(1) MAMC (cm)=midarm  circumference (cm)   −0.314×TSF (mm).

MAMC recently was validated as a correlate of lean body mass and associated with survival advantage in HD patients, especially in those with lower BMI [15].

### 2.4. Body Composition Analysis

Body composition was determined by body impedance analysis (BIA Nutriguard-M, Data-Input, Frankfurt, Germany). On the day of blood collection, patients underwent a BIA measurement at approximately 30 minutes postdialysis. BIA electrodes were placed on the same body side used for anthropometric measurements. The multifrequency technique was used. Recently, BIA was validated as reliable test for the estimation of total body fat percentage in maintenance hemodialysis patients [16]. Fat-free mass (FFM) was calculated by using the approach of Kyle et al. [17]. 

Fat mass and fat-free mass were standardized by squared height (m^2^) and expressed in kg/m^2^ as fat mass index (FMI) and fat-free mass index (FFMI), respectively. 

### 2.5. Laboratory Evaluation

Venous blood samples were collected from nonfasting patients before a midweek hemodialysis session in dry glass tubes and centrifuged, and the plasma was then aliquoted and kept frozen at −80°C before the assay. CBC, creatinine, urea, albumin, transferrin, and total cholesterol were measured by routine laboratory methods. Obestatin, leptin, TNF-*α*, and IL-6 levels were measured in plasma samples using commercially available enzyme-linked immunosorbent assay (ELISA) kits (R&D System, Minneapolis, MN, USA) according to the manufacturer's protocol. The mean minimal detectable level for obestatin was 0.41 ng/mL, for TNF-*α*—0.6 pg/mL, for IL-6—0.7 pg/mL and for leptin—7.8 ng/mL, respectively. The intraassay and inter-assay coefficients of variation were 3.5% and 5.6% for obestatin, 5.2% and 7.4% for TNF-*α*, 4.2% and 6.4% for IL-6, and 5.8% and 7.9% for leptin.

### 2.6. Statistical Analysis

Data are expressed as mean ± standard deviation (SD), median, and interquartile range (Q1 to Q3) for variables that did not follow a normal distribution and as percentages for categorical variables. 

Normally distributed continuous variables were compared between the two obestatin groups using a two-sided *t*-test and with chi-square tests for categorical variables. Since the dialysis vintage, leptin, TNF-*α*, and IL-6 levels were not normally distributed, median scores were used for comparisons using the nonparametric Mann-Whitney *U*-test.

Associations between two parameters were assessed using the Pearson correlation coefficients or Spearman rank order correlation coefficients, in the cases of skewed distribution of data. Logistic regression analyses were used to examine the nonlinear associations of obestatin with nutritional and inflammatory parameters in our patient population. 

To measure the differences between the variables in groups cross-classified by obestatin and TNF-*α*, a 2-factor MANOVA with the Wilks-lambda was used. 

Survival analyses were performed using the Kaplan-Meier survival curve and the Cox proportional hazard model. The univariate and multivariate Cox regression analyses are presented as HR; CI. The transfer to another center or the switching of a dialysis modality was regarded as censored information.

All statistical tests were two-sided, with a value for *P* < 0.05 defining significance. 

All statistical analyses were performed using SPSS software, version 16.0 (SPSS Inc., Chicago, IL).

## 3. Results

**Figure 1:**
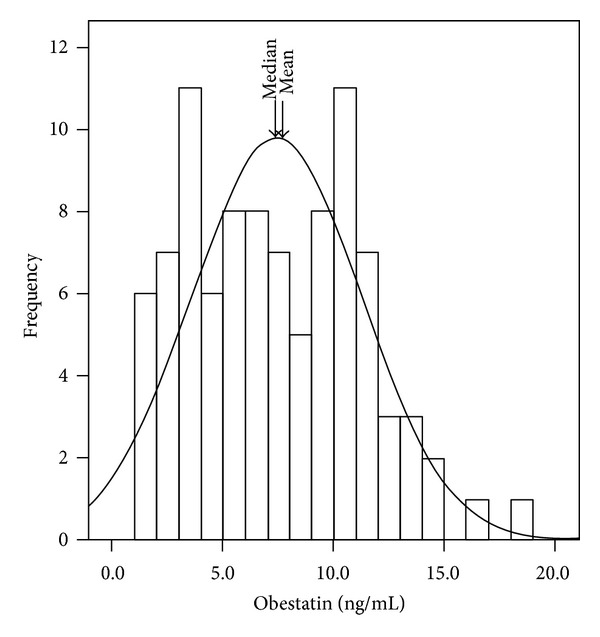
Histogram showing the distribution of serum obestatin concentration in the study cohort.

**Figure 2:**
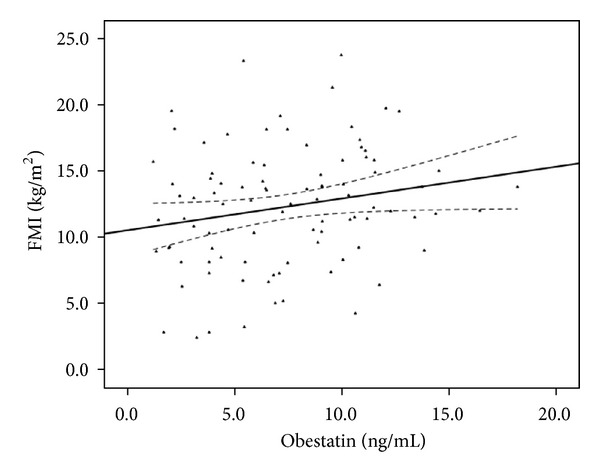
Correlations of obestatin and FMI of the study population at baseline: *r* = 0.210, *P* = 0.042. The solid line represents the regression line, and the dashed lines above and below solid line represent the 95% confidence interval.

For 94 prevalent HD patients at the start of the cohort, serum obestatin levels averaged (mean ± SD) 7.41 ± 3.8 ng/mL (median, 7.12 ng/mL; with interquartile range, 3.97–10.42 ng/mL).  Figure 1 shows the distribution of serum obestatin concentrations in the study population. Comparison of characteristics of the study subjects between the low and high obestatin groups, stratified by median of obestatin, are shown in Table 1. All characteristics; except gender, triceps skinfold thickness (TSF), midarm circumference (MAC), and fat mass index (FMI), are not significantly different between these two groups. No statistically significant differences were evident between the groups in the use of medications such as statins, aspirin, or angiotensin-converting enzyme inhibitors (data not shown). Among the various nutritional markers, only the FMI exhibited statistically significant linear association with obestatin (Figure 2). Each 5.0 ng/mL increase in serum obestatin level, controlled for age, gender, diabetes status, dialysis vintage, and history of cardiovascular disease, was associated with a lower daily energy intake (OR 2.12, 95% CI 1.01–4.47) by logistic regression analysis (data not shown). 

**Figure 3:**
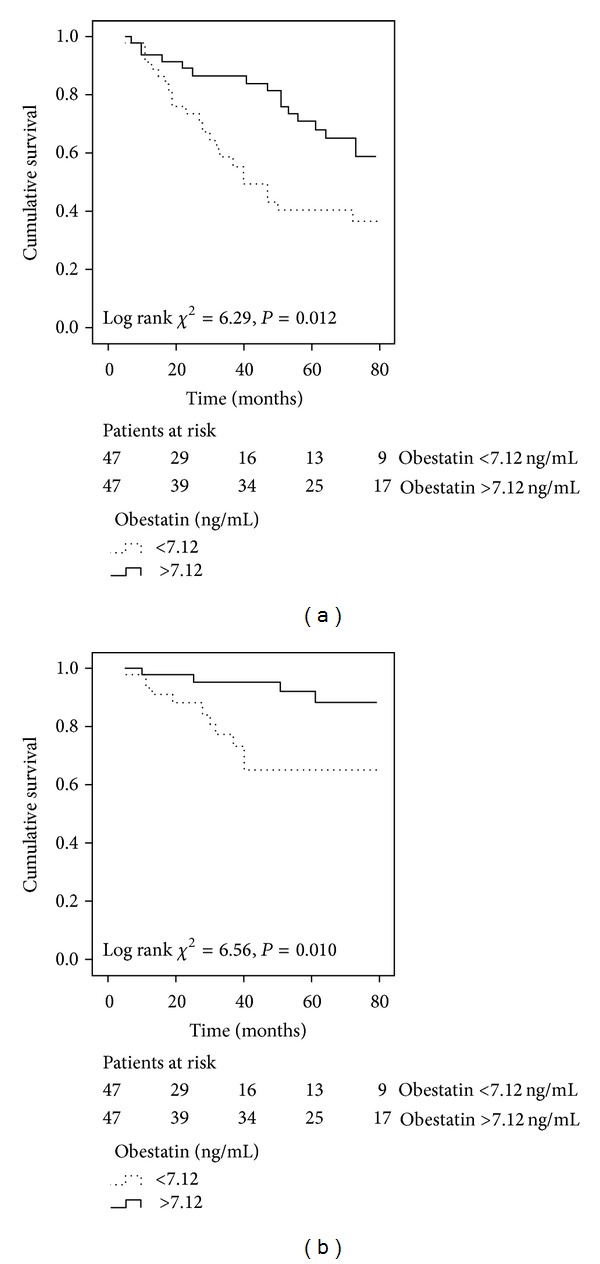
Kaplan-Meier survival curves of surviving patients comparing subgroups with baseline serum obestatin levels less or greater than the median value (7.12 ng/mL). (a) All-cause mortality, log-rank test *P* = 0.012. (b) Cardiovascular mortality, log-rank test *P* = 0.010.

Survival analysis was determined after a median follow-up period of 47 (19–75) months. During this period, 39 (41.5%) deaths occurred, of which 15 (38.5% of all deaths) were classified as cardiovascular deaths. The impact of obestatin levels on the outcome was studied by the Kaplan-Meier method. Patients with low obestatin levels, defined as lower than median level, had a worse all-cause mortality (Figure 3(a)) and cardiovascular mortality (Figure 3(b)). Of the 47 patients in the low obestatin subgroup, there was a total of 23 deaths (49%), of which 11 were related to cardiovascular causes (47.8%). In comparison, a total of 16 deaths (34%), of which only 4 were cardiovascular (25%), occurred in the same number of patients with high obestatin levels. Crude and adjusted Cox proportional hazard ratios (HRs) for mortality (Table 2) showed that patients from the low obestatin group had a significant crude HR (compared with patients with high obestatin) of 2.23 (95% CI: 1.17–4.24) for all-cause mortality and of 4.03 (95% CI: 1.27–12.76) for cardiovascular mortality that persisted after adjustment for age, gender, and diabetes status, but disappeared for cardiovascular mortality after further adjustments for history of past cardiovascular disease, dialysis vintage, and fat mass index. 

We then studied the effect of the modification of other important cytokines (such as TNF-*α*, leptin, and IL-6) on the relationship between low obestatin levels and clinical outcomes. The impact of low obestatin on all-cause and cardiovascular mortalities remained to be significant in stepwise multivariate Cox models (Table 3).

Furthermore, we tried to study the prognostic value of obestatin in the presence or absence of concomitantly increased levels of above mentioned cytokines. Statistical interaction analysis showed a departure from multiplicity of effects of low obestatin (less than median) with high TNF-*α* (above the median) levels. Crude Cox hazard ratio for all-cause mortality for the product termed obestatin X TNF-*α* was 3.01; with a 95% confidence interval, 1.47 to 6.15 (*P* = 0.003). No other statistically significant interactions between these cytokines and obestatin were found. 

**Figure 4:**
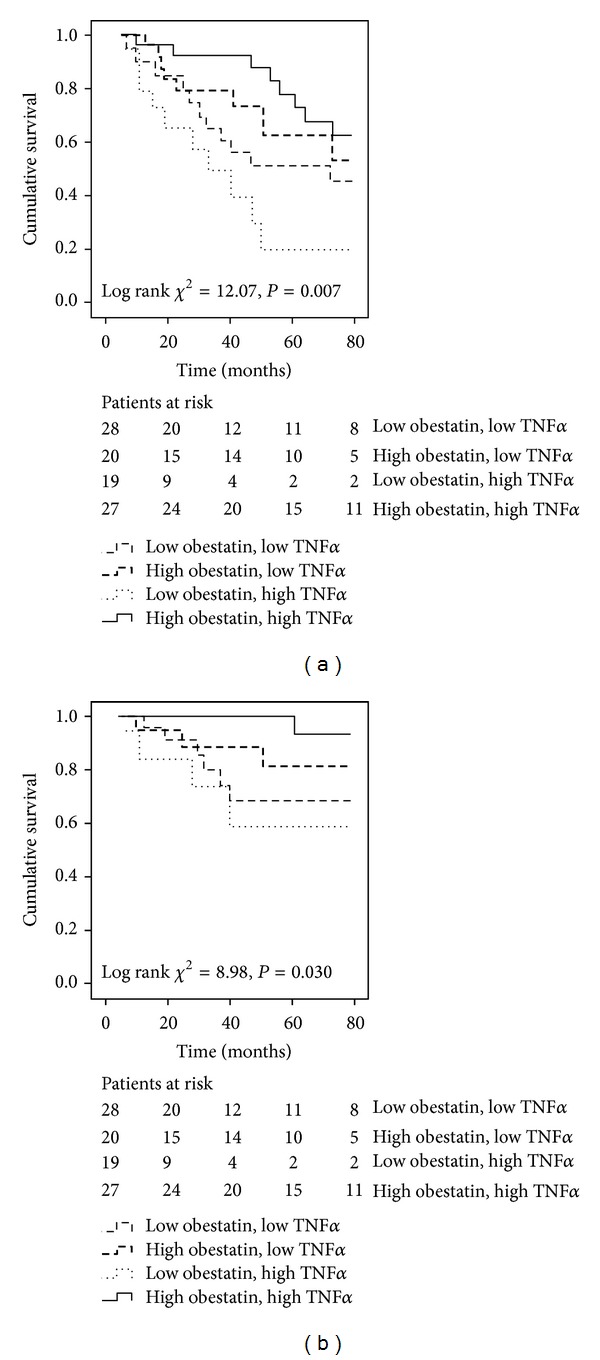
Kaplan-Meier survival curves of surviving patients comparing subgroups with baseline serum obestatin and TNF-*α* categories (obestatin below and above median (7.12 ng/mL) and TNF-*α* below and above median (17.75 pg/mL) values) cross-classified in 94 maintenance hemodialysis patients. (a) All-cause mortality, log-rank test *P* = 0.007. (b) Cardiovascular mortality, log-rank test *P* = 0.030. TNF-*α*: tumor necrosis factor-*α*.

In order to more closely study the interactions between obestatin and TNF-*α* on clinical outcomes in our cohort, different groups with high and low concentrations were established according to median obestatin and TNF-*α* levels and were cross-classified. The clinical and biochemical characteristics of the patients according to this categorization are detailed in Table 4. Patients with low obestatin levels had lower FMI and more men were in this group than in high obestatin group. Patients with high TNF-*α* also had more men than in the low TNF-*α* group. A significant obestatin X TNF-*α* interaction was found only for gender, since most patients from the low obestatin high TNF-*α* group were men. No other differences between the four groups, categorized according to obestatin and TNF-*α* levels, were found. Along with this, of the 19 patients in the low obestatin high TNF-*α* group, 11 patients died (57.9%) in contrast to 8 out of 27 patients (29.6%) that died in the high obestatin high TNF-*α* group. Moreover, only 1 cardiovascular death (3.7%) occurred in the latter group. Survival analysis for these four groups showed a negative impact of low obestatin but also a detrimental impact for concurrent high TNF-*α* values. Across the four obestatin-TNF-*α* categories, the group with low obestatin and high TNF-*α* exhibited a worse outcome in both, all-cause (log rank *χ* ^2^ = 12.07, *P* = 0.007) (Figure 4(a)) and cardiovascular (log rank *χ* ^2^ = 8.98, *P* = 0.030) mortalities (Figure 4(b)). Interestingly, the best clinical outcome was found in the high obestatin high TNF-*α* group. Further data adjustment for age, gender, diabetes status, and history of past cardiovascular disease, dialysis vintage, and FMI did not substantially affect these results (Table 5). 

## 4. Discussion

This study reports, for the first time in prevalent HD patients, an increased mortality risk for patients with low obestatin values. Moreover, to the best of our knowledge, to date, no study is available linking obestatin with mortality in any patient group or in the general population. Although this effect was modest and did not stand full adjustment for confounders in the case of cardiovascular mortality, the prognostic value of low obestatin on outcome seemed magnified in the context of its interaction with TNF-*α*. HD patients with high obestatin and high TNF-*α* concentration presented the lowest mortality risk, especially in cardiovascular-related mortality. In contrast, patients with low obestatin and high TNF-*α* concentration exhibited the worst outcome. Interestingly, whereas infection/sepsis-related mortality was not statistically different between these groups, only one cardiovascular death occurred in the high obestatin high TNF-*α* group. At the same time, the latter group of patients did not show any relevant demographic or clinical differences from the other groups classified by obestatin and TNF-*α* levels other than having a higher proportion of females. Similarly, basal obestatin levels in females were higher compared to that of males in a group of 321 normal weight and obese subjects [18] and 163 hypertensive adults participating in the Omni-Heart Trial [19]. Our findings with respect to the higher TNF-*α* levels in males compared to females (the whole high TNF-*α* group is considered here) are in agreement with those of Imahara et al. [20] but in contrast to those of Kalantar-Zadeh et al. [21] who did not find any difference in TNF-*α* levels between men and women in a cohort of 339 maintenance HD patients. As a consequence to our findings, gender was inserted in all multivariable models together with other potential confounders including FMI. 

Although clinical studies are still lacking, the available experimental literature provides some clues on the plausible mechanisms linking obestatin and obestatin-TNF-*α* interactions with cardiovascular diseases. Experiments in animal models and in vitro studies suggest that obestatin is a potential cardiovascular hormone, with various cardiovascular effects. These effects include vascular effects, expressed in significant vascular relaxation via specific activation of endothelium-dependent nitric oxide (NO) signaling [22], an endothelium protective effect in the presence of inflammation and therefore regulation of atherosclerosis [8], protective effects on myocardial ischemia-reperfusion injury [7] and, although still is controversial [23], a role in blood pressure regulation [24]. While the physiological role of obestatin is still largely unknown, these data may in part support the observed association in our population of low obestatin with mortality, especially from cardiovascular causes. 

In this context, it is of particular interest that high TNF-*α* appears as an aggravating factor of low obestatin-related mortality. The only available study linking obestatin-TNF-*α* interaction with cardiovascular disease comes from an in vitro study investigating effects of ghrelin and obestatin on the early key events of atherosclerotic processes such as monocyte adhesion to endothelial cells, binding of oxidized low-density lipoprotein (LDL), and acetylated LDL to macrophages [8]. In this study, obestatin dose dependently increased oxidized LDL binding to macrophages, a process that leads to foam cell formation, which is an essential step in atherogenesis [25]. However, in the presence of TNF-*α* (inflammation) obestatin inhibited VCAM-1 expression [8], suggesting that obestatin may modulate the processes participating in atherogenesis, but the effect can vary depending on the inflammatory condition. Applying this information to the results of our study, high obestatin may be assumed to exhibit antiatherogenic properties in the group of patients with high TNF-*α* levels and protect them from fatal cardiovascular events, whereas HD patients with high TNF-*α* but low obestatin levels are lacking such a protection and consequently have worse clinical outcomes. Two key messages should be adopted from this observation. First, an effect of modification by the interaction of obestatin (and probably of some similar molecules, participating in uremic milieu) with TNF-*α* may explain, at least in part, the existing conflicting data linking elevated circulating TNF-*α* levels to all-cause and cardiovascular mortalities in maintenance HD patients [26, 27]. Second, in interpreting the scant experimental evidence [8] and the results of our study, obestatin treatment theoretically may be useful only in HD patients with high TNF-*α* levels, whereas this treatment seems to be less effective or even harmful (proatherogenic) in HD patients with low TNF-*α* levels. Therefore, it is of utmost importance to find the most beneficial ratio in terms of the combination of the most important pro- and anti-inflammatory cytokines (which will probably include TNF-*α* and obestatin) that will be associated to the best clinical outcomes, before we decide to change their proportion by providing specific treatments. 

Finally, in contrast to the results of the earlier studies [19, 28], the cross-group comparisons did not confirm the association of serum obestatin level with nutritional markers (laboratory and body composition) in our study population. The associations between FMI and fat mass surrogates (TSF, MAC) with obestatin observed in our study were insignificant in multivariable models and therefore were not independent. In this aspect, our findings support the results of the recent studies in HD patients by Aygen et al. [9] and Mafra et al. [10]. Although we found nonlinear negative association between obestatin and dietary intake in our patients which is in line with some earlier data [3], a number of studies failed to reproduce the anorexigenic actions of obestatin [4]. Overall, nutritional characteristics of the two groups of HD patients, classified by median obestatin, did not differ in our study and consequently did not contribute to interpreting obestatin's prognostic usefulness. 

Some limitations of the present study should be considered. First, this study is based on a relatively small sample size, and our findings need confirmation in larger patient cohorts. Second, this study used only an observational approach, without manipulation of exposure factors, and therefore, no definitive cause-and-effect relationship can be derived for any of the risk factors analyzed. Third, the relatively low proportion of cardiovascular death may be due to its underestimation, because the causes of death were extracted from patient records and were not confirmed by autopsies. Fourth, samples were taken in nonfasting conditions, 1–3 h after a meal, because fasting blood samples were difficult to obtain from patients with diabetes and patients with an afternoon or night dialysis schedule. Although obestatin levels have been described to slightly decrease after meals in a healthy population [29], we are not aware of any study reported in the literature on the postprandial response of obestatin in end stage kidney disease patients. Dietary intake assessed by 3-day food records is another limitation of the study, as results can be subjective and incomplete. Finally, obestatin measurement in our study did not account for the parallel evaluation of ghrelin, another peptide which originates from the same preprohormone as obestatin and also has a number of beneficial cardiotropic effects: lowering of blood pressure, improving of endothelial function, regulation of atherosclerosis, protection from ischemia-reperfusion injury, and inhibition of myocardial cell apoptosis, as well as improving the prognosis of myocardial infarction and heart failure [6, 8]. However, association of ghrelin with clinical outcomes in HD patients has already been studied and reported recently [30]. Despite these limitations, the availability of a wide array of nutritional parameters applied, which include anthropometrics, biochemical markers, bioimpedance estimates of body composition, inflammatory biomarkers, and long-term followup, strengthens the study. 

## 5. Conclusions

This is the first report to show that low serum obestatin levels are linked to increased mortality risk in prevalent hemodialysis patients. In addition, novel interactions between obestatin and TNF-*α* associated with mortality risk, especially because of cardiovascular causes, were observed. The joint occurrence of high obestatin and high TNF-*α* seems to be associated with lower all-cause and cardiovascular mortalities risk, while coexistence of high TNF-*α* levels with low obestatin, in contrast, predicts the worst outcome. This behavior of obestatin in hemodialysis patients should be further elucidated. 

## Figures and Tables

**Table 1:** Comparison between subjects with lower and higher serum obestatin levels stratified by the median of obestatin in the study population (*n* = 94).

	Obestatin	Obestatin	*P* for trend
	≤7.12 ng/mL	>7.12 ng/mL	
	(*n* = 47)	(*n* = 47)	
Demographic and clinical variables			
Age (years)	64.6 ± 11.5	65.0 ± 11.6	0.88
Gender (men/women)^1^	68.1/31.9	55.3/44.7	0.023
Dialysis vintage (months)^2^	28.0 (12.0–47.0)	34.0 (20.0–54.0)	0.21
DM^1^	55.3	46.8	0.42
History of CV disease^1^	51.1	51.1	0.99
SBP (mm Hg)	139.4 ± 14.6	140.6 ± 20.3	0.75
DBP (mm Hg)	70.4 ± 11.7	69.6 ± 13.6	0.75
Kt/V	1.31 ± 0.25	1.31 ± 0.24	0.90
Dietary intake			
Energy intake (kcal/kg/d)	23.6 ± 5.5	22.2 ± 6.3	0.29
Protein intake (g/kg/d)	1.02 ± 0.20	0.95 ± 0.27	0.14
nPNA (g/kg/d)	1.07 ± 0.26	1.01 ± 0.29	0.30
Biochemical markers			
Albumin (g/L)	39.3 ± 2.9	39.9 ± 2.9	0.31
Creatinine (*µ*mol/L)	686.9 ± 194.5	691.3 ± 221.0	0.92
Cholesterol (mmol/L)	4.07 ± 1.0	4.22 ± 0.97	0.46
Transferrin (g/L)	1.69 ± 0.33	1.69 ± 0.32	0.98
TLC (×10^3^/mL)	1.70 ± 0.5	1.55 ± 0.4	0.15
Hemoglobin (g/L)	121 ± 11	121 ± 10	0.92
IL-6 (pg/mL)^2^	6.3 (3.3–11.6)	6.7 (3.3–10.9)	0.79
TNF-*α* (pg/mL)^2^	15.4 (9.0–32.9)	25.5 (11.7–35.5)	0.10
Leptin (ng/mL)^2^	16.2 (5.6–68.0)	23.0 (5.5–93.1)	0.49
Obestatin (ng/mL)	4.20 ± 1.7	10.6 ± 2.4	—
Anthropometric measurements			
BMI (kg/m^2^)	27.4 ± 5.2	28.7 ± 5.5	0.22
TSF (mm)	16.6 ± 6.2	21.3 ± 7.9	0.005
MAC (cm)	28.0 ± 3.7	30.3 ± 4.3	0.014
MAMC (cm)	22.8 ± 2.9	23.5 ± 2.7	0.22
Bioimpedance analysis			
ECW/TBW	0.39 ± 0.05	0.38 ± 0.05	0.56
FMI (kg/m^2^)	11.3 ± 4.7	13.3 ± 4.2	0.027
FFMI (kg/m^2^)	17.8 ± 2.1	17.7 ± 2.5	0.89
Phase angle (°)	4.9 ± 1.0	5.0 ± 1.0	0.48

Continuous variables are expressed as mean ± SD or as median with 25th to 75th percentile shown in parentheses in cases of nonnormally distributed data, and categorical variables are expressed as a percentage.

^1^Assessed by χ^2^ test.

^2^Compared by the nonparametric Mann-Whitney U test.

DM: diabetes mellitus; CV: cardiovascular; SBP: predialysis systolic blood pressure; DBP: predialysis diastolic blood pressure; nPNA: normalized protein nitrogen appearance; TLC: total lymphocyte count; IL-6: interleukin-6; TNF-*α*: tumor necrosis factor-*α*; BMI: body mass index; TSF: triceps skinfold thickness; MAC: midarm circumference; MAMC: midarm muscle circumference calculated; ECW/TBW: extracellular water to total body water ratio; FMI: fat mass index; and FFMI: fat-free mass index.

**Table 2:** Crude and adjusted Cox proportional hazard ratios for death in low obestatin (<7.12 ng/mL) group.

Model covariates	HR	95% CI	*P*
All-cause mortality			
(1) Crude	2.23	1.17–4.24	0.015
(2) 1 + age, gender, and DM	1.93	1.01–3.72	0.048
(3) 2 + vintage + CVD + FMI	2.04	1.02–4.07	0.043
Cardiovascular mortality			
(1) Crude	4.03	1.27–12.76	0.018
(2) 1 + age, gender, and DM	3.33	1.03–10.75	0.044
(3) 2 + vintage + CVD + FMI	3.20	0.94–10.84	0.062

All covariates included in regression models as continuous except for categorical variables.

Abbreviations: CI: confidence interval; HR: hazard ratio; CVD: cardiovascular disease in the past; DM: diabetes mellitus; and FMI: fat mass index.

**Table 3:** Multiple Cox regression analysis of obestatin, TNF-*α*, leptin, and IL-6 for predicting all-cause and cardiovascular mortalities.

	Model 1		Model 2		Model 3	
	HR (95% CI)	*P*	HR (95% CI)	*P*	HR (95% CI)	*P*
All-cause mortality						
Obestatin (<7.12 ng/mL)	2.44 (1.24–4.82)	0.010	2.47 (1.24–4.92)	0.010	2.46 (1.21–4.99)	0.013
TNF-*α* (<17.75 pg/mL)	0.75 (0.39–1.46)	0.40	0.75 (0.38–1.45)	0.39	1.16 (0.56–2.38)	0.69
Leptin (<16.7 ng/mL)			0.94 (0.49–1.78)	0.84	0.84 (0.44–1.62)	0.60
IL-6 (>6.4 pg/mL)					2.48 (1.20–5.13)	0.014
Cardiovascular mortality						
Obestatin (<7.12 ng/mL)	4.06 (1.23–13.39)	0.021	4.14 (1.24–13.85)	0.021	3.63 (1.08–12.27)	0.038
TNF-*α* (<17.75 pg/mL)	0.98 (0.33–2.86)	0.97	0.97 (0.33–2.85)	0.95	1.64 (0.52–5.17)	0.40
Leptin (<16.7 ng/mL)			0.90 (0.32–2.51)	0.83	0.90 (0.31–2.56)	0.84
IL-6 (>6.4 pg/mL)					2.73 (0.86–8.71)	0.089

Obestatin, TNF-*α*, leptin, and IL-6 were categorized according to their median levels, as indicated.

IL-6: interleukin-6; TNF-*α*: tumor necrosis factor-*α*; CI: confidence interval; HR: hazard ratio.

**Table 4:** Clinical and biochemical characteristics in 94 prevalent hemodialysis patients, according to obestatin and TNF*α* ^1^.

	Low TNF-*α* (*n* = 48)		High TNF-*α* (*n* = 46)		MANOVA^2^
	Low Ob. (*n* = 28)	High Ob. (*n* = 20)	Low Ob. (*n* = 19)	High Ob. (*n* = 27)	
Obestatin (ng/mL)	4.22 ± 1.8	10.2 ± 2.0	4.19 ± 1.6	10.89 ± 2.6	
Age (Y)	63.4 ± 12.5	67.2 ± 9.1	66.3 ± 10.1	63.3 ± 13.1	NS
Gender (men %)^3^	61	60	79	52	O, T, O × T
Log vintage (mo)	1.39 ± 0.34	1.42 ± 0.43	1.39 ± 0.48	1.54 ± 0.39	NS
DM (%)^3^	57.1	40	52.6	51.9	NS
CVD(%)^3^	46.4	60	57.9	44.4	NS
DEI (kcal/kg/d)	24.2 ± 5.2	21.1 ± 4.8	22.7 ± 6.0	23.3 ± 7.5	NS
DPI (g/kg/d)	1.06 ± 0.19	0.93 ± 0.17	0.98 ± 0.21	0.96 ± 0.34	NS
nPNA (g/kg/d)	1.14 ± 0.24	0.99 ± 0.26	0.99 ± 0.27	1.02 ± 0.37	NS
Albumin (g/L)	40.0 ± 2.7	40.3 ± 3.1	38.3 ± 2.9	39.7 ± 2.7	NS
Log IL-6 (pg/mL)	0.66 ± 0.49	0.73 ± 0.44	1.01 ± 0.41	0.86 ± 0.33	NS
Log leptin (ng/mL)	4.29 ± 0.52	4.10 ± 0.73	4.06 ± 0.76	4.39 ± 0.62	NS
BMI (kg/m^2^)	27.0 ± 4.6	28.4 ± 4.8	27.9 ± 6.2	29.0 ± 6.1	NS
FMI (kg/m^2^)	11.5 ± 4.3	12.3 ± 4.8	10.9 ± 5.3	14.1 ± 3.5	O
FFMI (kg/m^2^)	17.5 ± 2.2	17.8 ± 2.1	18.2 ± 2.1	17.6 ± 2.8	NS
ECW/TBW	0.38 ± 0.05	0.40 ± 0.04	0.40 ± 0.05	0.38 ± 0.06	NS

^1^The low obestatin or TNF*α* group was defined as obestatin < 7.12 ng/mL or TNF*α* < 17.75 pg/mL values below the median of distribution.

^2^Two-factor MANOVA. Significant (*P* < 0.05) effects are given for obestatin (O), TNF*α* (T), and the interaction obestatin with TNF-*α* (O × T).

Continuous variables that did not follow a normal distribution (dialysis vintage, interleukin-6, and leptin) were log transformed before their insertion in this model.

^3^Assessed by *χ* ^2^ test.

TNF-*α*: tumor necrosis factor-*α*; Ob: obestatin; DM: diabetes mellitus; CVD: cardiovascular disease in the past; DEI: daily energy intake; DPI: daily protein intake; nPNA: normalized protein nitrogen appearance; IL-6: interleukin-6; BMI: body mass index; ECW/TBW: extracellular water to total body water ratio; FMI: fat mass index; FFMI: fat-free mass index.

**Table 5:** Crude and adjusted all-cause and CVD-related mortality according to obestatin and TNF-*α* groups^1^.

Model covariates	Low TNF-*α*, low Ob.	*P*	Low TNF-*α*, high Ob.	*P*	High TNF-*α*, low Ob.	*P*	High TNF-*α*, high Ob.
	*n* = 28		*n* = 20		*n* = 19		*n* = 27
	HR (95% CI)		HR (95% CI)		HR (95% CI)		Ref
All-cause mortality^2^	12 (42.9%)		8 (40%)		11 (57.9%)		8 (29.6%)
(1) Crude	2.01 (0.82–4.92)	0.13	1.52 (0.57–4.05)	0.40	4.41 (1.74–11.15)	0.002	1.0
(2) 1 + age + sex + vintage	1.62 (0.65–4.02)	0.30	1.29 (0.48–3.47)	0.61	3.73 (1.43–9.72)	0.007	1.0
(3) 2 + DM + CVD + FMI	1.66 (0.63–4.41)	0.31	1.31 (0.48–3.54)	0.60	3.79 (1.44–10.0)	0.007	1.0
Cardiovascular mortality^2^	6 (21.4%)		3 (15%)		5 (26.3%)		1 (3.7%)
(1) Crude	7.76 (0.93–64.56)	0.058	4.41 (0.46–42.42)	0.20	13.78 (1.59–119.8)	0.017	1.0
(2) 1 + age + sex + vintage	6.13 (0.72–52.16)	0.096	3.78 (0.39–36.65)	0.25	10.55 (1.18–94.24)	0.035	1.0
(3) 2 + DM + CVD + FMI	5.62 (0.62–50.65)	0.13	3.46 (0.35–34.32)	0.29	9.92 (1.08–91.36)	0.043	1.0

^1^The group of patients who had high TNF-*α* (defined as TNF-*α* levels above median) and high obestatin (defined as obestatin levels above median) was used as a reference.

^2^Indicated as the number of deaths and percentage, expressed as a proportion of the total number of patients in the group.

The proportion of deaths was higher in the low obestatin group (in combination with either low TNF-*α* or high TNF-*α*) as assessed by *χ* ^2^ test (*P* = 0.002 for all deaths and *P* < 0.001 for CVD deaths).

All variables were included in regression models as continuous except for categorical variables.

CI: confidence interval; HR: hazard ratio; CVD: cardiovascular disease in the past; DM: diabetes mellitus; FMI: fat mass index; TNF-*α*: tumor necrosis factor-*α*; Ob.: obestatin.
